# Enhancing public health practice through a capacity-building educational programme: an evaluation

**DOI:** 10.1186/s12960-015-0024-4

**Published:** 2015-05-13

**Authors:** Preeti Negandhi, Himanshu Negandhi, Kavya Sharma, Sarah Wild, Sanjay Zodpey

**Affiliations:** Indian Institute of Public Health Delhi, Public Health Foundation of India, Plot No. 47, Sector 44, Institutional Area, Gurgaon, 122002 Haryana India; Centre for Population Health Sciences, University of Edinburgh, Medical School, Edinburgh, UK

**Keywords:** Public health, management, capacity-building, graduates, evaluation, knowledge, behaviour, practices

## Abstract

**Background:**

The Post-Graduate Diploma in Public Health Management, launched by the Govt. of India under the aegis of the National Rural Health Mission in 2008, aims to enhance the managerial capabilities of public health professionals to improve the public health system. The Govt. of India invested enormous resources into this programme and requested an evaluation to understand the current processes, assess the graduates’ work performance and identify areas for improvement.

**Methods:**

Quantitative telephone surveys as well as qualitative in-depth interviews were used. Graduates from the first three batches, their supervisors, peers and subordinates and faculty members were interviewed. Quantitative data were analysed using proportions, means and interpretative descriptions. Qualitative analyses involved transcription, translation, sorting, coding and filing into domains.

**Results:**

Of the 363 graduates whose contact details were available, 138 could not be contacted. Two hundred twenty-three (223) graduates (61.43% of eligible participants) were interviewed by telephone; 52 in-depth interviews were conducted. Of the graduates who joined, 63.8% graduates were motivated to join the programme for career advancement and gaining public health knowledge. The content was theoretically good, informative and well-designed. Graduates expressed need for more practical and group work. After graduating, they reported being equipped with some new skills to implement programmes effectively. They reported that attitudes and healthcare delivery practices had improved; they had better self-esteem, increased confidence, better communication skills and implementation capacity. While they were able to apply some skills, they encountered some barriers, such as governance, placements, lack of support from the system and community, inadequate implementation authority and lack of planning by the state government. Incentives (both monetary and non-monetary) played a major role in motivating them to deliver public health services. They suggested that states should nominate candidates expected to make a significant contribution to the health system, recognition from a relevant authoritative national body and need for a placement cell, especially for the self-sponsored candidates.

**Conclusions:**

A continuous mechanism for interaction and dialogue with the graduates during and after completion of the programme should be designed. This evaluation helped by providing inputs for refining the programme.

## Background

The delivery of quality health care is dependent on the knowledge and skills of public health personnel [1]. Competent public health professionals, with an in-depth understanding of health systems, can be expected to design, implement, monitor and evaluate health programmes, supervise the public health workforce, assess public health issues and develop appropriate strategies to address emerging challenges.

Although the National Rural Health Mission, now the National Health Mission (NHM), in India has supported initiatives for motivating and empowering health personnel within the public health system [2] since its launch in 2005, the public health system still faces a serious shortfall of trained public health managers. As a response, the Govt. of India, with the support of NHM, launched a 1-year residential teaching programme, the *Post Graduate Diploma in Public Health Management* (PGDPHM) in 2008, to train in-service public health professionals as well as self-sponsored candidates in public health management [3]. This programme was conceived as an inter-institutional partnership programme to enhance the public health management skills of public health professionals. An improvement in public health managerial skills addresses a felt need of public health systems within the country. Public health management training for public health professionals can be expected to improve their individual work performance as well as contribute towards an overall improvement across the public health system through better management. Candidates for the PGDPHM programme are selected either through government-supported nominations or are self-sponsored. The programme includes 8½ months of institution-based teaching, followed by 2½ months of field-based project work and 1 month of project writing and final evaluation. It is organized around a multi-disciplinary modular curriculum, focusing on public health (public health programmes, health systems reforms, behavioural sciences, etc.), management (finances, human resource, etc.) and analytical skills (epidemiology, statistics, etc.). The curriculum is competency driven and reflects the functions expected from a public health manager. The project work is an applied research component of the programme which exposes the students to “real-world” public health problems and encourages them to address these challenges through application of their knowledge and analytical skills in a community setting. After completing the programme, the government-nominated graduates return to the health system whereas the self-sponsored graduates look for employment in various government and non-government organizations. Details about the programme are available in a previously published paper on the subject [3].

Three hundred eighty-six (386) students graduated across the first three batches of the PGDPHM programme (four institutes in batch 1; seven institutes in batch 2; nine institutes in batch 3) [3]. The Govt. of India, through the NHM, has financially supported this programme. On its request, the PGDPHM programme was evaluated to understand the programme structure and processes and delivery of programme contents and assess the proximate outcomes related to the work performance of the PGDPHM graduates.

## Methods

### Study design

Quantitative telephonic surveys and qualitative in-depth interviews were conducted to gather data for the evaluation. Using two data collection methods aided the comparison of the findings from the quantitative survey and the qualitative interviews. While the quantitative methods facilitated the development of quantifiable information, the qualitative methods contributed to exploring subjective experiences of the study participants in greater detail.

### Study procedure

Approval for the study was obtained from the Institutional Ethics Committee at the Public Health Foundation of India, New Delhi. The sampling frame for this study included all the 386 graduates of the programme across three batches (2008–2009, 2009–2010 and 2010–2011). Partner institutes (nine institutes across India were offering this programme at the time of the evaluation) were requested to share the contact details of their graduates (telephone numbers, postal and e-mail addresses). A formal appointment was sought and informed consent was procured for participation into the study. Telephone interviews were conducted by either of two research associates hired for this activity between February and April 2012. The telephonic interview questionnaire was pretested on a group of students of another public health programme and revised based on their feedback. It included open- and close-ended questions regarding student experience during the programme including topics taught, appropriateness of the course curriculum in the context of the health system and new skills learnt. Graduates were also assessed for their performance at the workplace on parameters such as increase in knowledge and skills and better health-care delivery practices, applicability of these skills in the health system, facilitators and barriers in applying the skills based on what they learnt during the programme.

For the qualitative interviews, stratification criteria were used for the selection of graduates and their peers/supervisors/subordinates (Table 1). Topic guides for in-depth interviews were designed after pilot-testing. The themes for the in-depth interviews were related to how they were using their newly learnt skills with examples, what were the facilitators and barriers in application of these skills, what were the strengths and weaknesses of the programme in their opinion, etc. Fourteen in-depth interviews were conducted with graduates and twenty nine interviews were conducted with their peers/supervisors/subordinates. Nine interviews of faculty members/senior positions in institutes were also conducted. Several quality assurance measures were taken at various stages of the study.

**Table 1:** **Stratification criteria for selection of participants for qualitative interviews**

**Criteria for sampling/stratification**	**PGDPHM graduates (2008–2009, 2009–2010 and 2010–2011)**
Region	All states from which students have graduated in the first three batches were included. One state from each region (north, south, east, west and central) was randomly selected.
Distance from state headquarters	Only those graduates working at places that are accessible by road (within 5 h travel time) from the state headquarters were selected.
Years of work experience	<5 years or ≥5 years of experience in public health
Application category	Government-nominated or self-sponsored graduates
Gender	Both males and females were eligible as interviewees

### Data analysis

The responses from the telephone interviews were initially entered in a paper-based form, which were later entered in Epi-info 7. Responses were analysed as proportions and means for the close-ended questions and quotes and interpretative descriptions for the open-ended questions.

Data analysis of the qualitative interviews was done manually aided by computer-based software (Atlas.ti). The qualitative data, which were collected by recording the interview using a dictaphone, were transcribed and translated within the first 2 weeks of the interview. They were then sorted, coded and maintained in established files such as field files, mundane files and analytic files in an iterative process, comparing each interview with the previous interviews [4]. The codes were developed using the software. Codebooks were developed to analyse the transcribed data for each group of interviewees into specific codes (Table 2). The data were further analysed manually until specific domains were delineated. Common themes were generated to develop a conceptual model which represented the major study findings.

**Table 2:** **Codebooks used to categorize quotations from in-depth interviews for qualitative data analyses**

**Quotations of graduates**	**Supervisors/colleagues/subordinates**	**Institute heads/faculty members**
*General experience_course:* Description of the candidate’s general experience about the course.	*Skill_professional*: All those quotations related to knowledge and soft and technical skills.	*General experience_course*: Quotations which describe the institute heads’ and faculty members’ general experience about the course.
*Positive_teaching/learning*: Description of the candidate’s good experience about the teaching techniques, faculty and teaching aids.	*Skill_personal*: All those quotations related to communication, confidence, personality and time management	*Positives_curriculum*: Quotations which describe the institute heads’ and faculty members’ good experience about the curriculum.
*Shortcomings_teaching/learning*: Description of the candidate’s good experience about the teaching techniques, faculty and teaching aids.	*Application_acquired skills*: All those quotations related to application of learnt skills.	*Positives_teaching*: Quotations which describe the institute heads’ and faculty members’ good experience about the teaching.
*Shortcomings_curriculum*: Description of the candidate’s opinion on shortcomings about the course content and curriculum, material and course duration and dissertation.	*Barriers_utilization_acquired skills*: All those quotations related to barriers in acquired skills application.	*Shortcomings_curriculum*: Quotations which describe the institute heads’ and faculty members’ experience about the shortcomings in the curriculum.
*Positives_curriculum*: Description of the candidate’s good experience on course content and curriculum, material and course duration and dissertation.	*Suggestions*: All those quotations related to suggestions for improvement of the programme in the future.	*Infrastructure*: Quotations which describe the institute heads’ and faculty members’ experience about the infrastructure of the institute.
*Soft skills_personal*: Description of the candidate’s learning in terms of improved personal soft skills such as confidence, communication, attitude, thinking, computers and software.	*Opinion_PH strengthening*: All those quotations related to whether this course will be able to further strengthen the public health of the country	*Skills_personal*: Quotations which describe the institute heads’ and faculty members’ opinion about the candidate’s learnings in terms of improved personal soft skills such as confidence, communication, attitude, thinking, computers and software.
*Soft skills_professional*: Description of the candidate’s learning in terms of improved professional soft skills such as doctor patient relationship, human resource management, report writing and data analysis.	*Miscellaneous*: All those quotations not covered in the sub-headings mentioned above.	*Skills_professional*: Quotations which describe the institute heads’ and faculty members’ opinion about the candidate’s learnings in terms of improved professional soft skills such as doctor–patient relationship, human resource management, report writing and data analysis.
*Suggestions_course improvement*: Description of the candidate’s suggestions on course improvement.		*Application_acquired skills*: Quotations which describe the institute heads’ and faculty members’ opinion about the candidates’ attempt towards applying their learnings (successful/unsuccessful).
*Application_acquired skill*: Description of candidate talking about any attempt towards applying their learning (successful/unsuccessful).		*Barriers_skill implementation*: Quotations which describe institute heads’ and faculty members’ opinion about the barriers faced by the candidates in applying their learnings (successful/unsuccessful).
*Barriers_application_acquired skills*: Description of candidate talking about any barriers they faced in applying their learning (successful/unsuccessful).		*Opinion_PH strengthening*: Quotations which describe institute heads’ and faculty members’ talking about whether this course will be able to further strengthen the public health of the country.
*Suggestion_utilization_acquired skills*: Description of candidate giving suggestions about how their skills can be better utilized.		*Suggestions*: Quotations which describe institute heads’ and faculty members’ giving suggestions about how overall the programme can be further improved.
*Experience_job hunting* (private candidates): Description of candidate talking about placement cell and job hunting experiences.		*Miscellaneous*: Quotations which are not covered under above mentioned sub-headings will be put here.
*Experience_mixed group teaching:* Description of candidate talking about mixed-group teaching such as doctors and nurses, self-sponsored candidates and government-nominated candidates and candidates from different states.		
*Perceived importance_course*: Description of candidate talking about whether this course will be able to further strengthen the public health of the country.		
*Assistance_institute*: Description of candidate talking about some assistance from the institute in the form of workshops, reunions and refresher course.		
*Opinion infrastructure*: Description of candidate talking about the infrastructure of the institute.		
*Miscellaneous*: Quotations which are not covered under any of the above-mentioned sub-headings.		

## Results

**Figure 1:**
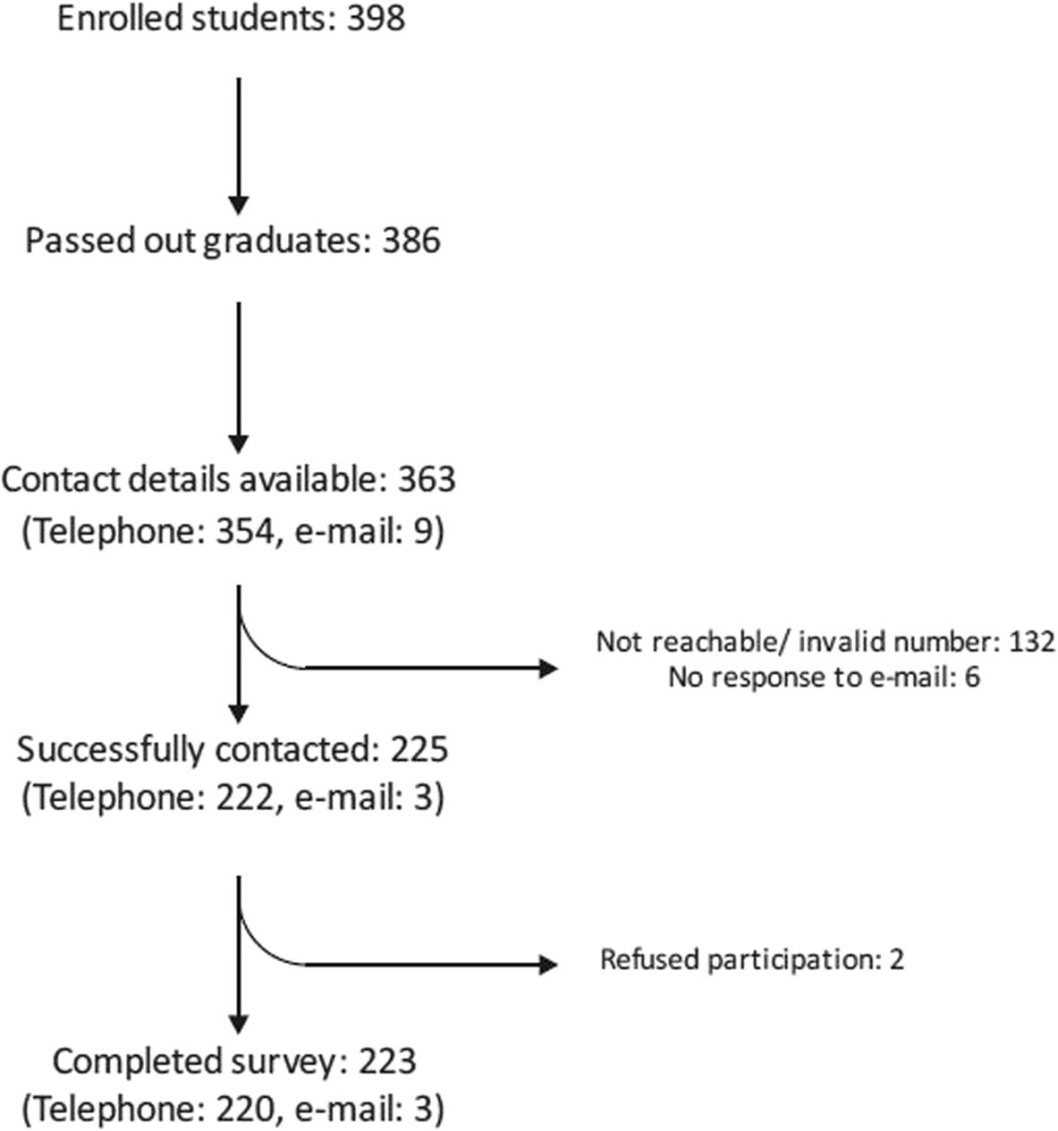
Flow diagram with details of telephonic interviews.

Of the total 386 graduates, we could procure the contact details of 363 graduates (354 Indians, 9 foreign nationals). Two hundred twenty-two (220) Indian graduates and three foreign graduates participated in the survey. The various stages of loss to follow-up are summarized in Figure 1, and responses were obtained from 61.43% of all graduates.

The profile of the respondents is summarized in Table 3 below. A large proportion of the participants (90.13%) were government-nominated doctors.

**Table 3:** **Participant profile: telephone survey**

**Profile descriptors**	**Frequency**
Mean age in years (SD)	42.26 (7.88)
Mean age government-nominated (SD)	42.43 (7.17)
Mean age self-sponsored (SD)	31.45 (5.57)
Mean work experience in years (SD)	15 (6.9)
*Sex—n (%)*	
Male	159 (71.30)
Female	64 (28.70)
*Batch wise distribution—n (%)*	
2008–2009	36 (16.14)
2009–2010	84 (37.67)
2010–2011	103 (46.19)
*Highest qualification—n (%)*	
MBBS	170 (76.23)
MD/MS	7 (3.14)
BAMS/BHMS/BUMS	7 (3.14)
B.Sc/M.Sc nursing	6 (2.69)
BDS/MDS	3 (1.35)
Others	20 (8.97)

**Figure 2:**
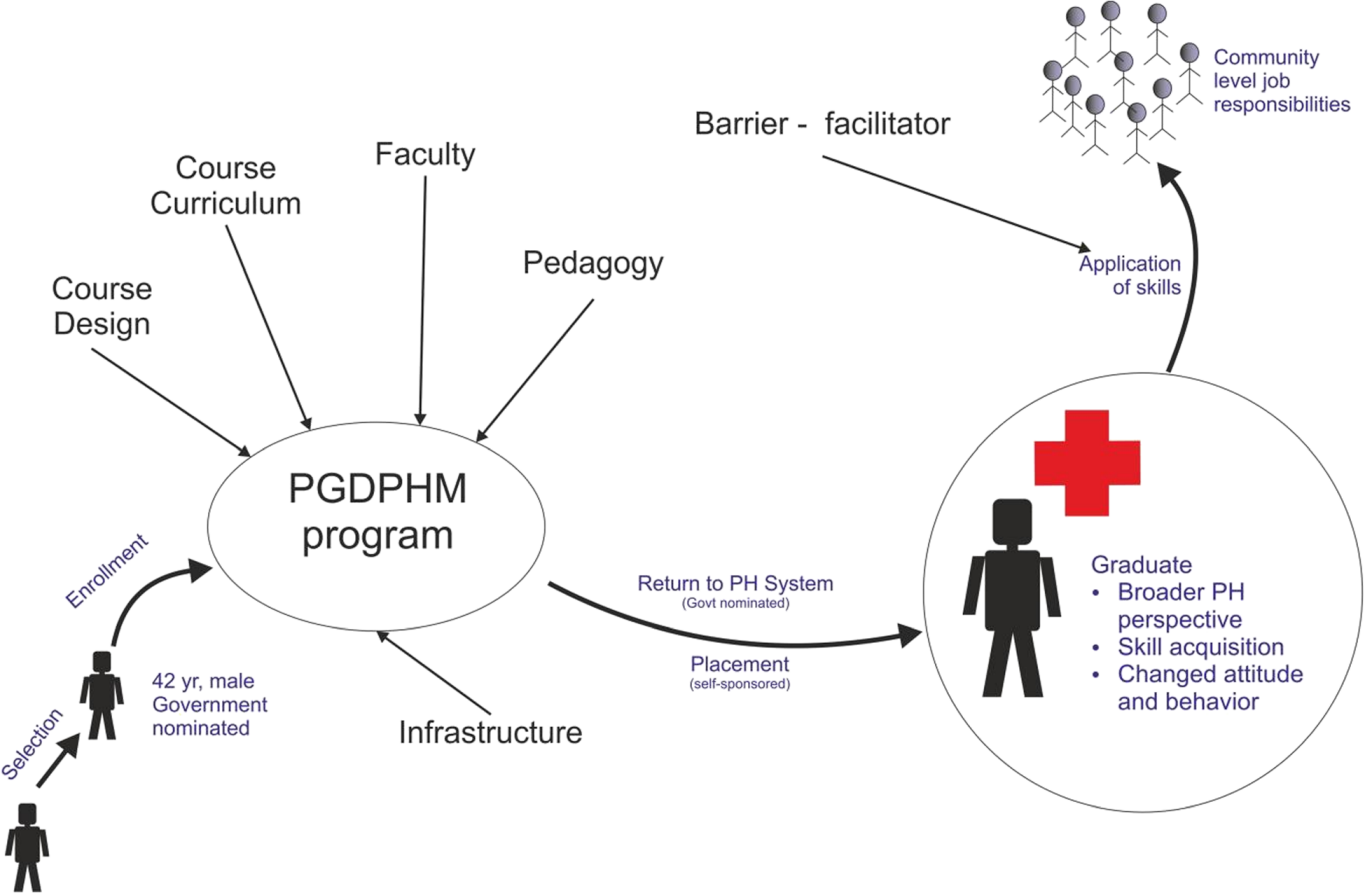
Conceptual model—aspects of the PGDPHM programme and its application in the health system.

Based on the responses of the telephonic surveys and the in-depth interviews, a conceptual model was developed to summarize the findings as Figure 2 depicts.

### Programme processes

The graduates’ main motivations to join the programme were for career advancement in public health (37.5%) and gaining knowledge of public health (26.3%), while some others who wished to continue their medical education needed a higher qualification in public health management or a good administrative job. A majority found the programme useful with respect to their day-to-day functioning of public health management and felt that they had acquired new knowledge and developed some new skills of public health management from this experience. While the vast experience of the government-nominated graduates was advantageous, there was a lack of eagerness from some of the senior candidates to join the programme at a late stage in their careers. They were interested in public health management; however, when asked how they anticipated the use of these competencies at their workplace, they stated that since they were in the late stages of their careers these skills and knowledge, although useful technically for the improvement of the health system, their contribution to the health system based on these competencies would be limited as against younger graduates who could spend a longer time in the health system after graduating the programme, thereby being able to contribute more to the system.

The institute heads and faculty members felt that this programme provided a unique focus on public health management and is deemed as a big attraction since it is offered by recognized institutes across the country.

Regarding the selection criteria of the candidates, a prominent response was that age of the candidate should be a consideration for enrolment. It was felt that younger nominated candidates and self-sponsored candidates extracted more learning from the programme. Institute heads and faculty members as well as supervisors, peers and subordinates suggested that states should select candidates transparently and in consultation with the institutes. A minimum 5 years of work experience in the health systems before enrolment was suggested.

The graduates openly discussed the course design, curriculum, faculty skills and pedagogy as well as academic infrastructure. Views regarding the programme duration were varied. While some felt the current duration (1 year) should be increased by 6 months to a year to allow for more time for project work, it was deemed appropriate by others. The institutional heads and faculty members, however, thought that the current programme duration was ideal. Almost all graduates agreed that the five core domains of public health, Epidemiology, Biostatistics, Behavioural and Social Sciences, Occupational and Environmental Health and Health Management, were taught in the programme. While 95 (43.38%) graduates found the Epidemiology and Biostatistics modules to be most useful, 45 (20.55%) felt that the Health Management module was the most useful. Twenty-six (11.87%) respondents felt that all modules were equally useful. The content was reported as being theoretically sound, well designed and informative. Some graduates stated that their project was helping them in their work, by aiding development of their research and writing skills, and giving them practical exposure. One of the institutional heads and faculty members also felt that the curriculum was

*…excellent and provides in-built flexibility in the course to add new things…*

They also felt that this programme was focused and covered topics extensively. They mentioned that the PGDPHM programme was able to provide the much needed management perspectives, especially for medical doctors.

During the in-depth interviews, the graduates stated that the teaching techniques used to deliver the PGDPHM programme were generally good and emphasized the good practical teaching skills of the Social Sciences department of one particular institute. The participants felt that the teaching was effective and was technology-based. The graduates had a positive view about learning as a mixed group as it encouraged exchange of ideas and perspectives among students who were from different academic backgrounds. The government-nominated candidates were benefited by the familiarity of the self-sponsored candidates with computers, while the government-nominated candidates shared their field experiences with the younger self-sponsored candidates.

There was consensus between the graduates and the institute members regarding the supportiveness and expertise of the faculty members teaching the programme. Good interaction with the faculty was reported by most graduates, who felt that this interaction should extend to project work and job hunting. While some graduates were happy with the overall infrastructure, some others felt that library space, Internet access within the hostel, academic space (at some institutes) and personal computers for their use were limited.

Another drawback of the programme was the inadequacy of printed learning materials. Availability of these would have assisted the graduates during the programme when the Internet was inaccessible or when they wanted to prepare notes, as also for reference after graduating the programme. Relatively older graduates and those from the health system strongly felt that the absence of easy notes and summarized text made the coursework difficult for them. Graduates also talked about the need to organize additional field visits within the programme. They perceived field visits to be very useful teaching methods alongside group discussions. Teaching practices that permitted the graduates to draw upon their work experience within health systems were preferred. It was suggested that the duration of some modules be readjusted or shortened. Institutional heads and faculty members also had similar views to express about certain modules, particularly Biostatistics. They talked about the programme containing too many modules, and also, management components, like logistics management, operational research and material management, were perceived to need further strengthening.

### Assessment of work performance

**Figure 3:**
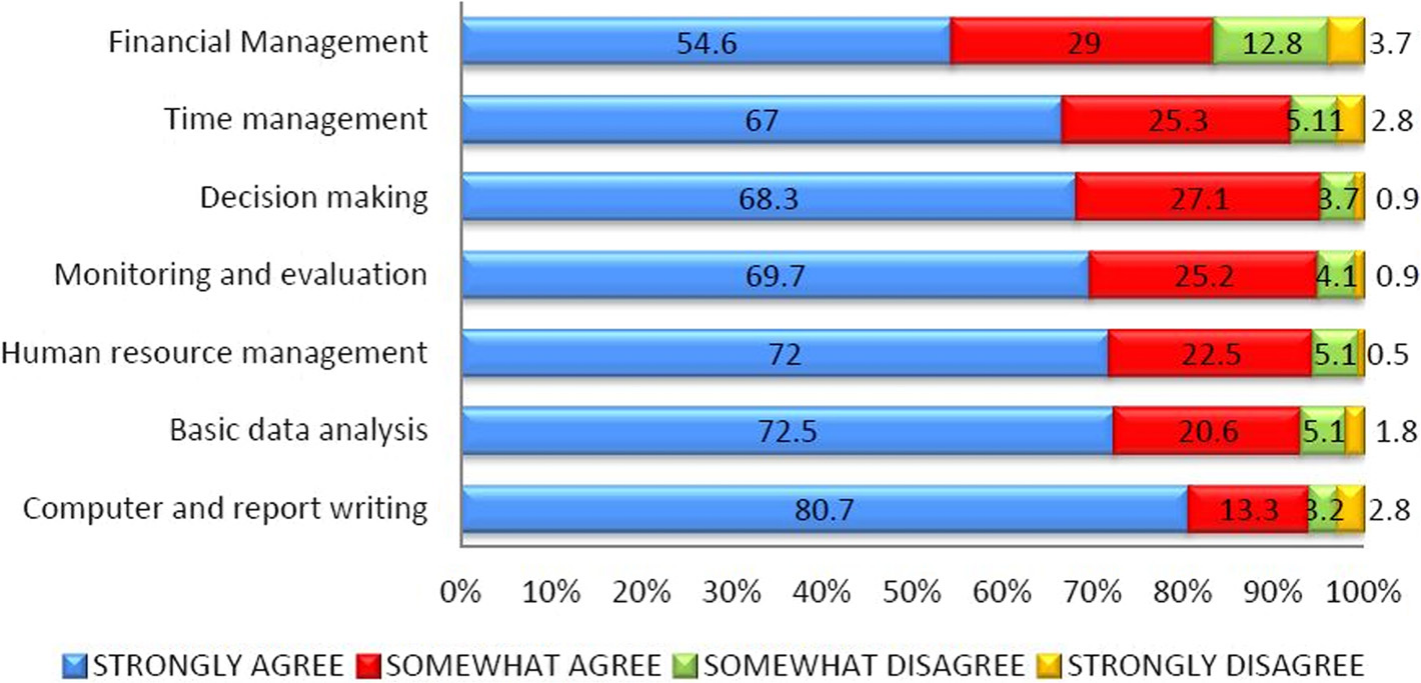
Perceptions of graduates about the newly acquired knowledge and skills during the PGDPHM programme.

The graduates felt that their public health vision had broadened and they were equipped with new skills after having completed the programme. Figure 3 outlines the skills acquired by them during the programme. For each skill, the graduates were asked telephonically whether they strongly or somewhat agreed or disagreed to having acquired it.

There were positive changes in their attitudes and behaviours. “…*I am now an improved man having a broader view of public health*” was the overall feeling one graduate expressed. They felt that there was a change in their perspective as well as their approach to their self-perceived job profile. They now believed in the concept of “ownership of work”, and their efficiency had also increased. This programme also helped some of them to aspire for further professional learning. Graduates felt that they were more confident, had better communication and language skills and had effective decision-making skills at the completion of the programme. Institute heads and faculty members reported opinions similar to the graduates on issues such as language development; better self-esteem, attitude and behaviour; increased confidence; better communication skills and thinking capacity in implementation. Similarly, the supervisors, peers and subordinates talked about improved communication and more confidence, innovative ideas, better time management, good doctor-patient relationship, leadership skills and a “go-getter” attitude among the graduates. Thus, the telephonic survey responses provided information on the essential technical skills, but the in-depth interviews additionally gave information related to specific context-oriented soft skills.

In the telephone survey, 123 (56.9%) graduates said that they were able to apply most of the skills acquired during the programme. During the in-depth interviews, they talked about using the newly acquired skills in different areas of their field work to the extent that their job provided (Table 4). They were using skills such as writing reports, giving technical inputs, supervising staff and effectively implementing programmes. The supervisors, peers and subordinates as well as institute heads and faculty members corroborated these responses. They discussed the usage of skills such as information, education and communication (IEC) activities, managerial role of the graduates cutting across various domains of their job profile, community intervention, staff management, monitoring and supervision, teaching (Table 4).

**Table 4:** **Skills predominantly used by the graduates at their workplace**

**(As reported by the graduates)**	**(As perceived by the supervisors, peers and subordinates)**
Data analysis	Increased computer usage
Usage of computer software	Improvement of PHC/infrastructure
Report making	Conducting training sessions regularly
Record keeping	Outbreak management
Logistics management (medicine)	Timely financial/logistics management
Outbreak/epidemic management	Monitoring and supportive supervision management
Patient counselling	Improved staff discipline/staff mindset changed
Health promotion activities in relation to national programmes	Development of programme guidelines
Problem-solving skills	Improvement in quality of health services
Supervisory skills	
Organization of events, including workshops	
Motivating staff	
Ability to give technical inputs to higher officials	
Prepare discussions from public health perspective	
Effective programme implementation	

The knowledge and skills collectively acquired by the graduates as reported by them and the other stakeholders represent the competencies that the programme content and curriculum are based upon. The graduates are expected to use these competencies during their work in the health system.

Table 5 shows that graduates were increasingly placed after graduating the PGDPHM programme at progressively higher level (community health centre/block, district, regional/state) within the health system.

**Table 5:** **Placement of the graduates in the health system (** ***n*** **= 223)**

**Placement within the health system**	**Before PGDPHM programme** ***n*** **(%)**	**After PGDPHM programme** ***n*** **(%)**
PHC	63 (28.2)	35 (15.7)
CHC/block	56 (25.1)	48 (21.5)
District	62 (27.8)	94 (42.2)
Regional/state	11 (4.9)	23 (10.3)
Any other	31 (13.9)	23 (10.3)

### Facilitators and barriers

When the graduates were questioned about the facilitators and barriers encountered while applying their skills, it was evident from most of the graduates’ views that incentives played a major role in keeping them motivated and enthusiastic for delivering their expected services. Some graduates expressed their disappointment on not getting any recognition in the system even after graduation, while 63% of graduates reported that no incentives were given to them in the health system after graduation. Barriers in skill application were issues of governance/politics and bureaucracy, inappropriate placements, frequent transfers, lack of support from the system/seniors/staff, inadequate powers to implement certain procedures, lack of infrastructure, lack of community support, contractual positions and lack of planning by the state government.

*There is no scope; we are entrapped in routine work right now. We do not have anything if we want to do something new…*

*We have learned about some financial things, which we can’t apply. We have been taught budgeting, but that much scope is not there in our day-to-day practice.*

Another very crucial aspect discussed was the need for the PGDPHM programme to be recognized by a relevant authoritative national body. Self-sponsored graduates reported that recognition/accreditation of the programme is essential for getting them a good job, whereas government-nominated graduates said that non-recognition of the programme was a major hindrance for them in getting promotions and relevant posts in the system.

There was a collective feeling among all the stakeholders of the study that the programme could contribute in strengthening the public health system of the country. Graduates felt that the programme provided the essential public health management skills which can help them in their day-to-day work. Some of them highlighted the need and importance of this programme by saying that it should be mandatory for all medical officers in the public health system.

Self-sponsored graduates were asked about their current placements as part of the mapping exercise, in which all the graduates who were employed were questioned about where they worked, their job profiles, etc., thus getting an idea of their employment positions across the country. Through this exercise, the utilization of their specific skills was mapped. Nineteen out of 22 interviewed (86.4%) self-sponsored graduates were currently employed. Of these, 42% are working in the government sector; others were involved with private organizations. During the in-depth interviews, the self-sponsored graduates stated that they had to struggle to find their jobs. Some of them stated that as newcomers it was difficult to find a job in the system even after graduating from the programme.

During both the telephonic surveys as well as the in-depth interviews, the self-sponsored graduates emphasized the need for a placement cell (a group which facilitates employments for the self-sponsored graduates), preferably within the institutes, to assist them in pursuing a job. Overall, a total of 195 (89%) said that they would recommend this course to others and 189 (86.3%) felt that it was relevant in their career advancement.

## Discussion

The PGDPHM programme has been operating since 2008 with a large majority of the graduates from the government sector. The graduates from the health system were less eager to join because they felt that they might not have enough opportunity to apply their skills and knowledge within their current job environment. On the other hand, the self-sponsored graduates, on average a younger group, were more motivated to gain knowledge and skills; this was also true for the young nominated graduates. The PGDPHM diploma provided an added qualification to these graduates. The reputation of the institutes offering this programme across the country was an additional incentive for enrolment of the candidates.

This study sampled all graduates of the PGDPHM programme for the telephone interviews. In-depth interviews of three different groups of stakeholders assisted data triangulation. Triangulation was done by collecting data from multiple sources, including graduates and their colleagues as well as teachers involved in implementing the programme at the institutes. Using multiple methods for data collection, including telephone survey and in-depth interviews, and comparing themes among the different methods of data collection helped in revealing a deeper understanding of the subject matter. Adoption of both manual and software-assisted analysis of the qualitative data further helped in minimizing researcher bias. However, despite multiple attempts to contact all graduates for the telephonic interviews, data were only collected from 61% of the graduates although respondents appear to be broadly representative of all graduates, given the available age and sex data of the study participants.

The current programme duration of 1 year was short from the perspective of some graduates. This programme was very likely their first formal academic exposure to public health and therefore an opportunity to equip themselves with public health skills since Masters in Public Health programmes are 1 or 2 years long, including project work, but most diploma courses are a year-long. Graduates also favoured a shortening of the modules which had minimal visible application in their day-to-day working in the health system. Previous studies suggest that service learning has been recognized as a useful teaching technique to link academic experience with community service [5]. This also explains their affirmative views on the inclusion and importance of project work in the PGDPHM curriculum.

It was largely observed that, while the opinions about the PGDPHM programme, in terms of the design, curriculum, faculty expertise, pedagogy, etc., were generally positive, there is scope for improvements in some areas, such as the course curriculum, inclusion of application-based teaching and provision of additional study resources. Institutes should design the course structure and learning to reflect the need of adult learners, while supporting the creation of adequate academic support structures. Adult-learning methods, to suit the needs of working professionals, should be relied upon during the course delivery. Modular content and their duration should be rationalized, and infrastructure should be strengthened.

The importance of interdisciplinary teamwork for better health care is recognized globally; this development should be fostered through inter-professional courses [6,7]. Mixed group teaching had a positive response among the graduates. Those who came from the state or district level got familiarized with the problems at the lower levels through those who came from these lower level health-care facilities. The graduates experienced a sense of mutual learning. Mixed-group learning gave them an opportunity to share their experiences with each other. The fact that this is an inter-institutional partnership being offered through multiple institutes across the country, it gives a fair and uniform opportunity to health personnel all over the country to gain knowledge and skills in the area of public health management, a felt need across all Indian states. The structure, content, overall execution and pedagogy of this programme are its selling points, and all these make the programme one of the best practices in public health education.

The study also explored the scope for application of the knowledge and skills acquired during the programme by the graduates at their respective workplaces in both the government and the private sector. A large majority of the graduates had limited or no knowledge of public health management at the time of enrolment into the programme but reported to have gained considerable knowledge in the subject during the programme. Some new soft skills and professional skills were also acquired, which had applicability in their work within health systems. The new knowledge and skills enhanced the capabilities of the graduates to contribute towards the health system. The change in their attitudes was also noticeable, as stated by their peers, supervisors and subordinates. They were eager to bring about a positive change in the functioning of the health system. However, there were some hurdles, mentioned by the graduates, which impeded the application of their skills and knowledge within health systems. Although more graduates have been placed at higher levels within the health system after completing the programme, they identified deterrents within health systems in the optimum use of their newly acquired skills.

The PGDPHM programme was largely looked upon by the enrolling candidates as an additional qualification for the advancement of their careers. The graduates expected the availability of a conducive environment to enable them to apply their learnings. Financial incentives and appropriate placements are essential motivational factors for candidates who are nominated by the state government. There were affirmative statements from some graduates who have been encouraged by their respective states. Initiatives taken by these states to promote their PGDPHM graduates could set an example for the other states. Additionally, recognition of this programme by relevant national bodies/organizations would boost the morale of the graduates as well as aid in facilitating their promotions and suitable placements.

For the self-sponsored candidates, the PGDPHM programme is a stepping stone towards career progression, and most of them look forward to appropriate placements after completing the programme. The current placements of some of these graduates show their willingness to take up not only private jobs but also to gain job experience in the public sector. The emphasis on the need for a placement cell with the assistance of the institutes is reasonable and warrants attention. Moreover, recognition of the programme would be beneficial to these graduates as well.

### Recommendations

On the whole, the PGDPHM programme was viewed as a beneficial knowledge-gaining platform and was recommended to be continued, taking into consideration certain areas with scope for improvement. Some of these need to be addressed collectively by the partner institutes in consultation with the Ministry of Health and Family Welfare, Govt. of India; some others may be addressed at the level of individual institutes. Candidates with a potential to make a significant contribution to the health system after graduation should be preferably nominated. States should also ensure that there is sufficient opportunity for the trained graduates to apply their knowledge and skills in the health systems after their return. As regards the programme structure, techniques that help students share their experience, latent skills and their competencies within the classroom and in the field should be predominantly adopted for the teaching–learning activities. The learning content of each module, its duration of teaching and its importance should reflect the perceived needs of the health systems and the candidates. Finally, state governments should design an enabling environment for utilizing the skills of the graduates optimally, some of which are mentioned in the “Suggestions to improve the utilization of the graduates’ skills acquired during the PGDPHM programme” section. A continuous mechanism for interaction and dialogue with the graduates, both during and after completion of the programme, should be designed. Efforts towards recognition/accreditation of the course should be pursued by the partner institutions with the Govt. of India and other relevant bodies.

### Suggestions to improve the utilization of the graduates’ skills acquired during the PGDPHM programme

- Creation of separate public health cadre
- Provision of incentives (monetary incentives, promotion)
- Better coordination between institutes and state health departments
- Secure permanent positions for better performance
- Appropriate managerial positions with authority
- Candidates interests should be assessed before joining PGDPHM programme
- Adequate staff to support initiatives undertaken

## Conclusions

The average age of the participants was 42 years; a large proportion (90.13%) was government-nominated doctors. Career advancement and an interest in continuing their medical education were the commonest motivating factors for joining this programme. The overall experience of the graduates about the programme was positive. They found the programme useful with respect to their day-to-day functioning and felt that they had learnt many new things and acquired some new skills during the programme. During the surveys and in-depth interviews, the graduates discussed various aspects of the course and offered suggestions for further improvement in these domains and in the academic infrastructure. Further, inappropriate placements, lack of support from the system, insufficient authority and lack of community support influenced their ability to apply all their learning in their respective workplaces.

The recommendations of this evaluation cover suggestions to revise some of the programme content and its delivery by the institutes, as well as an initiative to continue dialogue between the states and the institutes for appropriate and optimum utilization of the training received by the graduates.

The overall positive responses from not only the graduates of the programme but also the other relevant stakeholders at the teaching institutes as well as within the health system where these graduates have returned for work would help the Govt. of India take a positive decision about continuing this programme for other health personnel across the country and reap its benefits optimally for improvement of the health systems. It would be worthwhile to conduct a follow-up study to look at the sustainability of the programme in terms of the knowledge and skills acquired by them and their long-term career growth within the health system. It would also be fruitful to assess the influencers for the incorporation of public health management knowledge in undergraduate medical curricula and in situated practices throughout professional training for the purpose of its subsequent translation into practice.
